# Prolonged Response to an IGF-1 Receptor Antibody in a Patient with Metastatic Castration Prostate Cancer with Neuroendocrine Differentiation

**DOI:** 10.7759/cureus.426

**Published:** 2015-12-22

**Authors:** Julie N Graff, George V Thomas, Celestia S Higano, Tomasz M Beer

**Affiliations:** 1 Knight Cancer Institute, Oregon Health & Science University; 2 Seattle Cancer Care Alliance, University of Washington

**Keywords:** igf-1, castrate-resistant prostate cancer

## Abstract

The androgen receptor is the main therapeutic target that has been successfully exploited through direct inhibition to extend survival of patients with metastatic castration-resistant prostate cancer (mCRPC). We present a patient who participated in a Phase II study of an antagonist antibody to insulin-like growth factor 1 receptor (IGF-1R) in men with mCRPC and experienced over five years of stable disease. His disease was rapidly progressing before exposure to the antibody and resumed its aggressive behavior following discontinuation of therapy, strongly supporting the attribution of his stable disease to IGF-1R inhibition. His pre-treatment biopsy exhibited increased protein expression of IGF-1R (and its downstream effector, phosphorylated-S6). Consequently, agents that target IGF-1R may provide profound and durable responses in a subset of patients and upfront molecular selection may enable us to identify those most likely to benefit.

## Introduction

There are six agents that prolong survival in metastatic castration-resistant prostate cancer (mCRPC). Several small molecule and antibody-based targeted agents, such as those used for colon cancer (EGFR), lung cancer (EGFR) and renal cell carcinoma (VEGF, mTOR), show modest activity in Phase II studies of prostate cancer, but the level of activity has not been sufficient to substantially improve clinical outcomes in patients.

Prostate cancer growth depends on androgen receptor (AR) signaling. The classical mechanism of AR signaling involves ligand binding in the cytoplasm, receptor dimerization, and translocation to the nucleus, followed by transcriptional regulation of target genes. Clearly, the AR has a central role in prostate cancer progression, but there are other elements that contribute to prostate cancer aggressiveness, both through the AR and independently of AR signaling. Insulin-like growth factor (IGF-1) binds to its receptor insulin-like growth factor-1 receptor (IGF-1R) and promotes prostate cancer growth through MAPK and PI3K signaling [1-2]. IGF-1R expression is enhanced on prostate cancer cells, and higher levels of IGF-1R are associated with both the risk of developing prostate cancer and the risk of developing metastatic disease [3-4]. IGF-1R has been shown to activate the PI3K/Akt and may stimulate nuclear translocation of the AR [5]; therefore, it is a tantalizing target in prostate cancer.

Here, we describe a man with mCRPC who experienced stable disease for over five years on a clinical trial using an antibody against IGF-1R, and we attempt to understand the underlying biology of his tumor that may have enabled him to benefit from a therapy that did not benefit a large fraction of patients treated.

Informed patient consent was obtained. The patient was also involved in a total of three different protocols approved by the Oregon Health and Science University (OHSU) Knight Cancer Institute. They are as follows:

Phase II Single Arm, Open-Label Study of IMC-A12 in Asymptomatic, Chemotherapy-Naive Patients with Metastatic Androgen-Independent Prostate Cancer (IRB #3584) (The main clinical trial in which he received the IGF-1R.)

Radiologically Guided Biopsies of Metastatic Castration-Resistant Prostate Cancer to Identify Adaptive Mechanisms of Resistance (IRB #9204) This is the clinical trial he participated in where the biopsies were done.

Master Protocol for Cancer Research Specimen Bank and Database (IRB #2816). This is the one used to request his original tissue and stain for the IGF-1, etc.

## Case presentation

Thirty years ago, at the age of 47 years, our patient was diagnosed with metastatic prostate cancer to the bone when he presented with acute back pain. Remarkably, 3 mg per day of diethylstilbestrol (DES) taken orally maintained disease stability for 24 years, at which time his PSA began to rise despite a castrate level of testosterone (28 ng/dl). His bone scan showed metastatic disease to the thoracic spine, sternum, and multiple ribs. His prostate gland was biopsied for the first time after the development of mCRPC and revealed the features consistent with high-grade prostate adenocarcinoma, Gleason’s Grade 4 + 4 = 8.

Flutamide was added to the DES and then discontinued for diarrhea. It was replaced with nilutamide, which transiently controlled his disease. Eight years ago, his PSA began to climb. His cancer did not respond to bicalutamide, 50 mg per day orally, with DES. He discontinued DES and started a luteinizing hormone-releasing hormone (LHRH) agonist with bicalutamide, with a brief and modest decrease in serum PSA. His bicalutamide was discontinued without a withdrawal response.

A nuclear bone scan revealed three rib lesions, multiple lesions in the sternum, and one lesion at the T12 pedicle as well as uptake in the cervical and thoracic spine concerning for cancer involvement. His CT scan showed three sub-centimeter lung lesions concerning for involvement. He volunteered for a Phase II, open-label, clinical trial of cixutumumab (IMC-A12), a fully human IgG1 monoclonal antibody that specifically inhibits IGF-1R binding and signaling in men with an asymptomatic mCRPC (NCT00520481). At the time of enrollment, his PSA was 21 ng/ml.

**Figure 1:**
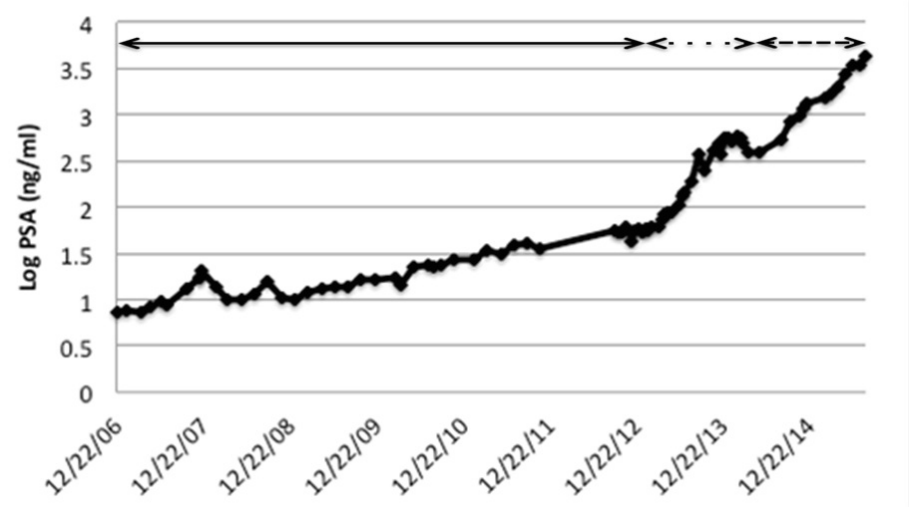
PSA Trend Log-transformed PSA trend of our patient on IGF-1R (solid line), abiraterone/prednisone (dotted line) and radium-223 (dashed line).

In this clinical trial, subjects received cixutumumab, 10 mg/kg every two weeks or 20 mg/kg every three weeks, until intolerance or progression. Our patient was treated with 10 mg/kg every two weeks. He experienced several expected dermatologic toxicities beginning just after the first dose when he had nine days of Grade 1 dermatitis. Eight months into treatment, cixutumumab was held for Grade 2 nonpalpable purpura. Evaluation by a hematologist did not reveal a hematologic cause for his purpura, as he had normal coagulation factors and a very mild thrombocytopenia to 114 K/mm^3^. Within four weeks of holding the study medication, his purpura resolved. Upon restarting, he experienced a facial rash evaluated by his dermatologist and deemed likely study-related. He also developed brittle fingernails, Grade 1. The purpura ranged from Grade 0-1 and did not lead to any other dose delays. Both dermatologic toxicities persisted until he was taken off of the study for frank progression of cancer on his bone scan after over five years of treatment with cixutumumab. During the treatment, he did not have a reduction of lesions on radiographs or clear PSA decrease. His PSA nadired at 10 ng/ml and slowly – and with a fluctuating pattern - increased over the course of five years without radiographic progression, and it was 186 ng/ml when he was taken off study (Figure 1). Nuclear medicine bone scans, obtained every two months during the first year, every three months during years three and four, and then every four months, showed stable disease until five new lesions were detected after five years. CT imaging did not show any soft tissue progression.

After the study, he received abiraterone with prednisone, but his cancer rapidly progressed. His PSA rose from 373 ng/ml to 510 ng/ml over four months time, and he had more than five new metastatic lesions at the three-month nuclear medicine scan that were confirmed on subsequent imaging. He is currently being treated with radium-223 and has declined chemotherapy.

**Figure 2:**
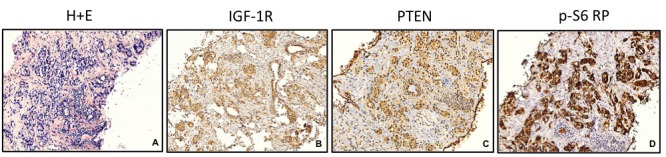
IGF-1R, PTEN and p-S6 RP protein expression in pre-treatment prostate biopsy Hematoxylin and eosin stain of prostate biopsy with prostate adenocarcinoma. IGF-1R, PTEN and p-S6 RP protein expression by immunohistochemistry demonstrates strong expression compared to stroma (40x).

With his written consent, formalin-fixed paraffin embedded prostate biopsy tissue obtained after the development of mCRPC *and prior* to the initiation of IGF-1R therapy were immunostained for IGF-1R (Cell Signaling Technologies #14534). In addition, since the PI3K-mTOR oncogenic pathway is downstream of growth stimulatory signals from IGF-1R, we examined the levels of PTEN and phosphorylated-S6 (Cell Signaling Technologies #9188 and #4858, respectively)*. *IGF-1R and phosphorylated-S6 protein expression were scored as follows: 0 for no staining in the tumor, 1+ for weak staining, and 2+ for moderate to strong staining, compared to adjacent benign glands and stroma. PTEN staining was scored according to a previously established scale of 0 to 2, which has been shown to be highly consistent [6]. Tumor cells were graded as 2+ if their staining intensity is equal to that of the adjacent benign cells, as 1+ if their staining was diminished relative to the benign cells, and as 0 if staining intensity is undetectable in the tumor cells and is present in the benign cells. The tumor cells had 2+ staining for the IGF-1R, phosphorylated-S6, and PTEN, suggesting increased expression of IGF-1R and its downstream substrate p-S6 in the presence of intact PTEN expression (Figure 2).

Subsequently, he joined a non-therapeutic biomarker study, which included biopsies of metastatic lesions pre- and post-second generation hormone therapies. Thus, we were able to biopsy an osseous metastatic deposit after progression on IGF-1R and prior to treatment with abiraterone/prednisone. All tissues on the biomarker study are examined for AR by FISH (Fluorescence In Situ Hybridization) and the presence of PTEN by IHC, and his tissue showed AR amplification and intact PTEN protein expression. Next, using a next generation sequencing platform developed at our institution, we screened his biopsy for actionable mutations that included RTKs, catalytic and regulatory components of PI3K, and PTEN. Interestingly, this screening did not reveal any mutations in* PI3K *components or *PTEN. *Unfortunately, there was insufficient tissue to stain for IGF-1R and phosphorylated-S6. Indeed, his sustained response might be related to lack of certain mutations in his tumor, e.g. intact PTEN and P53.

## Discussion

Despite presenting with metastatic disease at a young age, following an extraordinarily durable response to DES, this patient had an extended period of disease stability with a high quality of life during treatment with an IGF-1R antibody that did not produce similar results in other trial participants. Notably, the requirement that PSA not be used to change therapy enabled him to benefit from therapy and us to detect the clinical benefit of IGF-1R inhibition.

Phase II studies of IGF-1R in prostate cancer have shown some promise, but there are currently no Phase III studies of IGF-1R inhibitors (www.ClinicalTrials.gov). Our patient’s study enrolled 31 patients and revealed disease control lasting more than six months in nine (29%) in the every-two-week dosing and three (30%) in the every-three-week dosing [7]. The longest disease control, other than our patient, was four years, so there was a subset of patients who clearly benefited. The median time to composite progression (i.e. first time to unequivocal evidence of tumor progression by RECIST or bone scan, new skeletal-related events, symptomatic progression if no measurable disease, or other clinical events attributable to prostate cancer in the opinion of the investigator) was 3.8 months. The most common adverse events (AEs) were fatigue (39%; 7% Grade 3) and hyperglycemia (23%; 16% Grade 3). Of the five patients with Grade 3 hyperglycemia, four required insulin and none required cixutumumab discontinuation. Other cixutumumab-related Grade ≥ 3 toxicities were fatigue, thrombocytopenia, leukoencephalopathy, and Grade 5 pneumonia.

Serial biopsies in men with mCRPC have led to the identification and characterization of the neuroendocrine differentiation that frequently accompanies resistance to the second-generation androgen signaling inhibitors, enzalutamide, and abiraterone [8]. Re-examination of his first biopsy confirmed Gleason 4+4 adenocarcinoma. His cancer also returned with positive p-S6, potentially a marker of downstream IGF-1R activity. His second biopsy (post-IGFR antibody and pre-abiraterone) revealed an intact PTEN and AR amplification. Interestingly, PTEN and IGF-1R both act on PI3K, and there is some evidence that loss of PTEN together with high levels of IGF-1R are detrimental in men with prostate cancer [9], but this does not apply directly to our patient since his tumor exhibited intact PTEN protein expression and there was no evidence of PTEN mutation or loss of heterozygosity.

## Conclusions

We hypothesize that the rarely seen prolonged response to IGF-1R blockade seen in this patient may be related to the increased expression of IGF-IR and its downstream substrate (as documented by strong staining for IGF-IR phosphorylated p-S6 in tumor cells compared to adjacent benign glands). This may have implications for a precision medicine approach and use of cixutumumab in prostate and other cancers where the IGF-IR pathway is important.
